# Face Mask Use and Control of Respiratory Virus Transmission in Households

**DOI:** 10.3201/eid1502.081167

**Published:** 2009-02

**Authors:** C. Raina MacIntyre, Simon Cauchemez, Dominic E. Dwyer, Holly Seale, Pamela Cheung, Gary Browne, Michael Fasher, James Wood, Zhanhai Gao, Robert Booy, Neil Ferguson

**Affiliations:** University of New South Wales School of Public Health and Community Medicine, Sydney, New South Wales, Australia (C.R. MacIntyre, H. Seale, J. Wood, Z. Gao); Children's Hospital at Westmead, The University of Sydney, Sydney (C.R. MacIntyre, P. Cheung, R. Booy, G. Browne); Imperial College London, London, UK (S. Cauchemez, N. Ferguson); Westmead Hospital, Sydney (D.E. Dwyer); The Wentworth Division of General Practice, Sydney (M. Fasher)

**Keywords:** Masks, respiratory viruses, influenza, infection control, community, household, research

## Abstract

Many countries are stockpiling face masks for use as a nonpharmaceutical intervention to control virus transmission during an influenza pandemic. We conducted a prospective cluster-randomized trial comparing surgical masks, non–fit-tested P2 masks, and no masks in prevention of influenza-like illness (ILI) in households. Mask use adherence was self-reported. During the 2006 and 2007 winter seasons, 286 exposed adults from 143 households who had been exposed to a child with clinical respiratory illness were recruited. We found that adherence to mask use significantly reduced the risk for ILI-associated infection, but <50% of participants wore masks most of the time. We concluded that household use of face masks is associated with low adherence and is ineffective for controlling seasonal respiratory disease. However, during a severe pandemic when use of face masks might be greater, pandemic transmission in households could be reduced.

Many countries are stockpiling face masks for use as nonpharmaceutical interventions to reduce viral transmission during an influenza pandemic. We conducted a prospective cluster-randomized trial comparing surgical masks, non–fit-tested P2 masks, and no masks in prevention of influenza-like illness (ILI) in households. During the 2006 and 2007 winter seasons, 286 exposed adults from 143 households who had been exposed to a child with clinical respiratory illness were recruited. Intent-to-treat analysis showed no significant difference in the relative risk of ILI in the mask use groups compared with the control group; however, <50% of those in the mask use groups reported wearing masks most of the time. Adherence to mask use was associated with a significantly reduced risk of ILI-associated infection. We concluded that household use of masks is associated with low adherence and is ineffective in controlling seasonal ILI. If adherence were greater, mask use might reduce transmission during a severe influenza pandemic.

## Abstract 2

Mask use is associated with low adherence, but adherent mask users are significantly protected against seasonal disease.

Highly pathogenic avian influenza virus A (H5N1) continues to spread globally, posing a serious human pandemic threat. In the event of an influenza pandemic or other emerging respiratory disease such as severe acute respiratory syndrome (SARS), it is likely that antiviral drugs and vaccines will be in short supply or that delivery could be delayed. Therefore, nonpharmaceutical interventions such as mask use, handwashing, and other hygiene measures or school closure might be effective early control strategies. In contrast to pharmaceutical interventions, little is known about the effectiveness of nonpharmaceutical interventions in the community. A recent analysis gives estimates of the effect of school closure (*1*), and several prospective, randomized controlled trials of handwashing have been published (*2**–**11*). However, clinical trial data on the ability of face masks to reduce respiratory virus transmission in the community are limited to 1 published prospective trial, which showed lack of efficacy (*12*). In addition, adverse effects of wearing masks (particularly respirators) may affect compliance and effectiveness (*13**–**15*). Despite the lack of quantitative evidence, many countries have included recommendations in their pandemic plans on the use of face masks (*16**–**18*). We present the results of a cluster-randomized household study of the effectiveness of using face masks to prevent or reduce transmission of influenza-like illness (ILI).

## Methods

A prospective, cluster-randomized trial of mask use in households was conducted during the 2 winter seasons of 2006 and 2007 (August to the end of October 2006 and June to the end of October 2007) in Sydney, Australia. Enrollment in the study was restricted to households with >2 healthy adults >16 years of age; the adults had known exposure within the household to a child with fever and respiratory symptoms. Suitable households were identified at a pediatric health service comprising the emergency department of a pediatric hospital and a pediatric primary care practice in Sydney, New South Wales, Australia. The study protocol was approved by the local institutional review board.

### Randomization and Intervention

**Figure 1:**
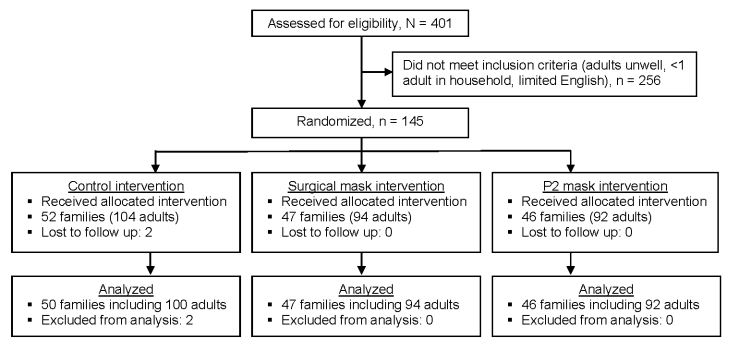
Flow diagram of recruitment for the prospective cluster-randomized trial, Sydney, New South Wales, Australia, 2006 and 2007 winter influenza seasons.

Participating households were randomized to 1 of 3 arms by a secure computerized randomization process: 1) surgical masks (3M surgical mask, catalogue no. 1820; St. Paul, MN, USA) for 2 adults, to be worn at all times when in the same room as the index child, regardless of the distance from the child; 2) P2 masks (3M flat-fold P2 mask, catalogue no. 9320; Bracknell, Berkshire, UK), for 2 adults, to be worn at all times when in the same room as the index child, regardless of the distance from the child; and 3) a control group (no masks used). The P2 masks used have an almost identical specification as N95 masks used in the United States (*19*). According to New South Wales Health guidelines, pamphlets about infection control were provided to participants in all arms. Study participants and trial staff were not blinded, as it is not technically possible to blind the mask type to which participants were randomized. However, laboratory staff were blinded to the arm of randomization. Figure 1 shows the flow diagram for the trial as suggested by CONSORT guidelines (*20*).

### Recruitment and Follow-up

Children 0–15 years of age seeking treatment at pediatric health services with fever (temperature >37.8^o^C) and either cough or sore throat were identified by an electronic triage system. Parents or primary caregivers were approached in the waiting room, and that household was invited to join the study if all of the following criteria were satisfied: 1) the household contained >2 adults >16 years of age and 1 child 0–15 years of age; 2) the index child had fever (temperature >37.8^o^C) and either a cough or sore throat; 3) the child was the first and only person to become ill in the family in the previous 2 weeks; 4) adult caregivers consented to participate in the study; and 5) the index child was not admitted to the hospital.

If eligibility criteria were satisfied, adults from the household were enrolled in the study. Enrolled adults and any siblings of the index child were then evaluated for respiratory symptoms and signs (fever, history of fever or feeling feverish in the past week, myalgia, arthralgia, sore throat, cough, sneezing, runny nose, nasal congestion, headache). If any of these symptoms were present, the family and household were excluded. Sociodemographic and medical information including influenza vaccination history (both the index child and participating adults) was obtained using a researcher-administered questionnaire. Medication use was also recorded. The index case-patient had combined nasal (each nostril) and throat swabs collected for multiplex reverse transcription–PCR (RT-PCR) testing. The household was randomized to 1 of the 3 arms, allocated the appropriate mask type, and educated about infection prevention. Formal fit testing of the P2 masks was not performed, but information pertaining to the correct method for fitting and disposing of the masks was provided. Over the next week, participants were contacted by telephone daily to determine if symptoms had developed and to record adherence to mask use throughout the day.

Each household was supplied with a thermometer to measure the temperature of symptomatic adult participants twice daily. If study staff determined that a participant had developed respiratory disease symptoms at follow-up, a home visit was conducted on the same day and the participant was swabbed and tested for respiratory viruses (see methods described below). Symptomatic participants were then followed up daily for 2 weeks.

Because all respiratory pathogens share similar transmission mechanisms—aerosol, droplet, and fomite spread (although the relative role of these factors may vary among different viruses and in different clinical situations)—we deliberately considered a broad definition of clinical cases consistent with a wide range of common respiratory viruses. Respiratory viruses detected in the study included influenza A and B, respiratory syncytial virus (RSV), adenovirus, parainfluenza viruses (PIV) types 1–3, coronaviruses 229E and OC43, human metapneumovirus (hMPV), enteroviruses, and rhinoviruses.

Adherence to face mask use was specifically monitored during each household follow-up. Measuring adherence and reasons for nonadherence is critical for evaluating the efficacy of mask use for reducing treatment and for providing practical advice on future use of face masks. Exit interviews with participants in the surgical mask and the P2 mask arms were conducted to gain further insights into adherence.

### Sample Collection and Laboratory Testing

Rayon-tipped, plastic-shafted swabs were inserted separately into each participant’s nostrils and pharynx, placed into viral transport media, and transported immediately to the laboratory or stored at 4^o^C if transport was delayed. Nose and throat swabs of index children and adult participants with symptoms of respiratory illness were tested by using nucleic acid and a series of multiplex RT-PCR tests (*21*) to detect influenza A and B and RSV, PIV types 1–3, picornaviruses (enteroviruses or rhinoviruses), adenoviruses, coronaviruses 229E and OC43, and hMPV.

### Case Definition

To include the broadest possible spectrum of clinical syndromes occurring among enrolled adults (*22*), during follow-up we defined ILI by the presence of fever (temperature >37.8°C), feeling feverish or a history of fever, >2 symptoms (sore throat, cough, sneezing, runny nose, nasal congestion, headache), or 1 of the symptoms listed plus laboratory confirmation of respiratory viral infection. The choice of a relatively broad clinical case definition was dictated by our interest in interrupting transmission of a broad range of respiratory viruses. Laboratory-confirmed cases during the follow-up were defined by the presence of >1 of the symptoms listed above plus laboratory detection of a respiratory virus.

### Study Outcomes and Analysis

The primary study outcomes in enrolled adults were the presence of ILI or a laboratory diagnosis of respiratory virus infection within 1 week of enrollment. Given that we demonstrated some dual infections and that there may be a variable sensitivity of RT-PCR for different respiratory viruses, we included all incident infections in adults (by clinical case definition and laboratory testing) in the analysis. We also measured the time from recruitment to infection. Causal linking of the outcomes of ILI and adherence to use of face masks required consideration of the timing of both.

Analysis of primary outcomes was by intention to treat. We performed a multivariate Cox proportional-hazards survival analysis to study secondary outcomes and determine how time lag from recruitment to infection of a secondary case-patient was affected by explanatory covariates (*23*). Gaussian random effects were incorporated in the model to account for the natural clustering of persons in households (*24*). The day of infection was reconstructed from the day of symptom onset under the assumption that the incubation period was 1–2 days. To account for exposures that occurred before recruitment, the time when survival analysis started was defined as the maximum value between the day of recruitment minus the incubation period and the start of illness in the index case. (For example, assume a household recruited on day 0 and an incubation period of 2 days. If illness in the index case began on day –3, then the survival analysis began on day –2; if illness in the index case began on day –1, then the survival analysis began the same day.)

The following variables were included in the models: daily adherence to use of P2 or surgical masks, number of adults in the household, number of siblings in the household, and index case <5 years of age. This analysis was performed using the survival package of the statistical software R (www.r-project.org). Comparisons among groups were made with the Fisher exact test for categorical variables. A 2-sided p value <0.05 was considered significant.

### Power Analysis

Assuming a secondary attack rate in exposed adults of 20% and an intraclass correlation coefficient of 30%, we estimated that 94 adults would be needed in each arm of the study to show efficacy of >75% of P2 or surgical masks at 80% power and with a p value of 0.05. Our efficacy estimate was a conservative assumption based on observational data for the combined effects of all mask types during the SARS epidemic in Hong Kong (*25*).

## Results

### Study Population

We recruited 290 adults from 145 families; 47 households (94 enrolled adults and 180 children) were randomized to the surgical mask group, 46 (92 enrolled adults and 172 children) to the P2 mask group, and 52 (104 enrolled adults and 192 children) to the no-mask (control) group. Two families in the control group were lost to follow-up during the study. Characteristics of the families who participated are shown in Table 1, with no significant differences noted among the 3 arms.

**Table 1:** Demographic characteristics of each household by arm of randomization in the study, Sydney, New South Wales, Australia, 2006 and 2007 winter influenza seasons

Variable	Control group, no. (%), n = 50	Surgical mask group			P2 mask group	
		No. (%), n = 47	p value		No. (%), n = 46	p value
Living arrangement						
Reside in house	38 (76)	32 (68)	0.39		33 (72)	0.64
>4 persons in house	13 (26)	18 (38)	0.20		19 (41)	0.11
>3 adults in house	8 (16)	11 (23)	0.36		12 (26)	0.23
Demographics						
Caucasian race*	28 (56)	20 (43)	0.18		17 (37)	0.06
Both adults work	28 (56)	25 (53)	0.78		27 (59)	0.79
Smoker in house	12 (24)	12 (26)	0.86		4 (9)	0.046
Index child fully immunized	45 (90)	45 (96)	0.28		39 (85)	0.44
Index child attends childcare	37 (74)	34 (72)	0.85		27 (59)	0.11
Influenza vaccination						
Index child	1 (2)	1 (2)	0.97		0	0.34
1 adult vaccinated	2 (4)	2 (4)	0.95		0	0.17
Duration of child sickness†	4	5			4	
Siblings reporting illness	3 (6)	1 (1)	0.34		0	0.09

*Information relates to the participating adult interviewed. †Median no. days.

Samples were collected from 141 children; respiratory viruses were detected in 90 (63.8%) children. In 79 (56.0%) of 141 cases, a single pathogen was detected: influenza A in 19/141 (13.5%); influenza B in 7/141 (4.9%); adenoviruses in 7/141 (4.9%); RSV in 5/141 (3.5%); PIV in 8/141 (5.5%) (PIV-1 in 1/141 [0.70%]; PIV-2 in 2/141 [1.4%]; PIV-3 in 5/141 [3.5%]); hMPV in 8/141 (5.7%); and coronavirus OC43 in 3/141 (2.1%). Other viruses detected included picornaviruses in 22/141 (15.6%): rhinoviruses in 11/22 (50.0%); enteroviruses in 5/22 (22.7%) (enterovirus 68 in 1/5 [20.0%] and others in 4/5 [80.0%]); and uncharacterized nonsequenced picornaviruses in 6/22 (27.0%). An additional 11 children (7.8%) had dual or co-infection: 4 (2.8%) with adenovirus and rhinovirus, 2 (1.4%) with rhinovirus and coronavirus; and 1 each with influenza A and enterovirus, influenza A and PIV-2, influenza A and rhinovirus, RSV and enterovirus, and adenovirus and hMPV.

### Adherence

**Figure 2:**
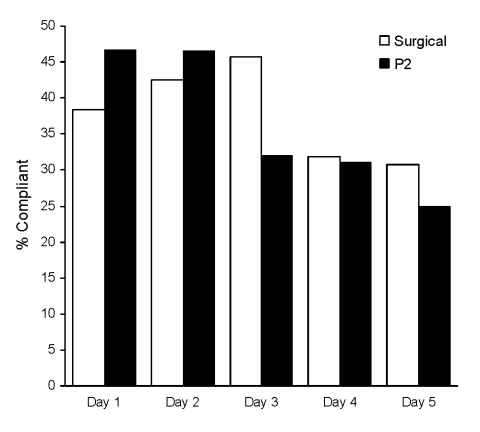
Compliance with mask use by day over 5 consecutive days during the study, Sydney, New South Wales, Australia, 2006 and 2007 winter influenza seasons.

Characteristics of the adherent versus nonadherent participants who were recruited are shown in Table 2; no significant differences were noted between the 2 groups except for the presence of >3 adults in the household. On day 1 of mask use, 36 (38%) of the 94 surgical mask users and 42 (46%) of the 92 P2 mask users stated that they were wearing the mask “most or all” of the time. Other participants were wearing face masks rarely or never. The difference between the groups was not significant (p = 0.37). Adherence dropped to 29/94 (31%) and 23/92 (25%), respectively, by day 5 of mask use (Figure 2).

**Table 2:** Characteristics of adherent versus nonadherent mask wearers in the study, Sydney, New South Wales, Australia, 2006 and 2007 winter influenza seasons *

Variable	Fully adherent mask users, no. (%), n = 30	Nonadherent mask users, no. (%), n = 156	p value
Living arrangement			
Reside in house	22 (73)	108 (69)	0.66
>4 persons in house	11 (37)	64 (41)	0.66
>3 adults in house	3 (10)	43 (28)	0.04
Demographics			
Caucasian race†	10 (33)	29 (19)	0.07
Working adult	22 (73)	118 (76)	
Smoker in house			
Daily handwashing	14 (45)	54 (34)	0.21
Use of soap when handwashing	13 (43)	65 (42)	0.87
Index child fully immunized	15 (50)	69 (44)	0.56
Index child attends childcare	6 (20)	51 (33)	0.17
Influenza vaccination			
Index child	0	1 (0.5)	0.66
Adult 1	0	2 (1)	0.53
Adult 2	0	2 (1%)	0.53
Median days of child sickness	5	5	
Siblings reporting illness	0	1 (0.5)	0.66

*Adherence to mask use and handwashing measured by daily self-reports and exit interviews. †Information relates to the participating adult interviewed.

Table 3 shows reported problems with mask use. There were no significant differences in difficulties with mask use between the P2 and surgical mask groups, but >50% reported concerns, the main one being that wearing a face mask was uncomfortable. Other concerns were that the child did not want the parent wearing a mask and the parent forgot to wear the mask. Additional comments made by some included that the mask did not fit well and that it was not practical to wear at meal time or while asleep. Some adults wore the mask during the day but not at night, even though the sick child was sleeping beside them in their bed.

**Table 3:** Problems with face mask use reported by participants in the study, Sydney, New South Wales, Australia, 2006 and 2007 winter influenza seasons

Reported problem	Surgical mask users, no. (%), n = 94	P2 mask users, no. (%), n = 92	p value
None	46 (49)	42 (46)	0.66
Uncomfortable	16 (17)	14 (15)	0.74
Forgot to wear it	8 (9)	8 (9)	0.96
Child did not like it	6 (6)	8 (9)	0.55
Other	18 (19)	20 (22)	0.66

### Intention-to-Treat Analysis

ILI was reported in 21/94 (22.3%) in the surgical group, 14/92 (15.2%) in the P2 group, and 16/100 (16.0%) in the control group, respectively. Samples were collected from 43/51 (84%) sick adults, with respiratory viruses isolated in 17/43 (40%) sick adults. Viral pathogens were isolated from 6/94 (6.4%) in the surgical mask group, 8/92 (8.7%) in the P2 group, and 3/100 (3.0%) in the control group. In 10/17 laboratory-positive cases, the same respiratory virus was isolated in the adult and the child (surgical, 3/94; P2 group, 5/92; and control, 2/100). In 2 cases, the adult was the only person with a laboratory-confirmed virus (1 each from the P2 and surgical groups); in the remaining 5 adults, the virus detected in the child differed from that in the adult (surgical, 2; P2 group, 2; and control group, 1). No dual infections were detected in the adults. Intention-to-treat analysis by households and by participants showed no significant difference between the groups (Table 4).

**Table 4:** Intention-to-treat analysis used in the study*

Data	Control group, no. (%)	All masks				Surgical masks				P2 masks		
		No. (%)	RR (95% CI)†	p value†		No. (%)	RR (95% CI)†	p value†		No. (%)	RR (95% CI)†	p value†
By house	n = 50	n = 93				n = 47				n = 46		
ILI	12 (24)	25 (27)	1.12 (0.62–2.03)	0.84		15 (32)	1.33 (0.70–2.54)	0.50		10 (22)	0.91 (0.43–1.89)	0.81
By individual	n = 100	n = 186				n = 94				n = 92		
ILI	16 (16)	33 (18)	1.11 (0.64–1.91)	0.75		19 (20)	1.29 (0.69–2.31)	0.46		14 (15)	0.95 (0.49-1.84)	1
Laboratory confirmed infections												
Influenza A	0	3 (2)				1 (1)				2 (2)		
Influenza B	0	1 (0.5)				0				1 (1)		
RSV	1 (1)	1 (0.5)				0				0		
hMPV	0	0				0				0		
Adenoviruses	0	2 (1)				0				2 (2)		
PIV‡	1 (1)	1 (0.5)				1 (1)				0		
Coronaviruses*§*	1 (1)	0				0				0		
Rhinoviruses	0	5 (3)				3 (3)				2 (2)		
Enteroviruses	0	0				0				0		
Picornoviruses	0	1 (0.5)				0				1 (1)		
Total	3 (3)	14 (8)	2.51 (0.74–8.5)	0.19		6 (6)	2.13 (0.55–8.26)	0.32		8 (9)	2.90 (0.79–10.6)	0.12

*RR, relative risk; CI, confidence interval; ILI, influenza-like illness; RSV, respiratory syncytial virus; hMPV, human metapneumovirus; PIV, parainfluenza virus. †Reference group is the control group. ‡Types 1–3; 229E/OC43. §Types 1–3.

### Risk Factors for ILI

Under the assumption that the incubation period is equal to 1 day (the most probable value for the 2 most common viruses isolated, influenza [*21*] and rhinovirus [*26*]), adherent use of P2 or surgical masks significantly reduces the risk for ILI infection, with a hazard ratio equal to 0.26 (95% CI [confidence interval] 0.09–0.77; p = 0.015). No other covariate was significant. Under the less likely assumption that the incubation period is equal to 2 days, the quantified effect of complying with P2 or surgical mask use remains strong, although borderline significant; hazard ratio was 0.32 (95% CI 0.11–0.98; p = 0.046). The study was underpowered to determine if there was a difference in efficacy between P2 and surgical masks (Table 5).

**Table 5:** Estimates of hazard ratios for ILI in the study*

Variable	Global effect of mask use			Effect per mask type	
	Hazard ratio (95% CI)	p value		Hazard ratio (95% CI)	p value
1-d incubation period					
Adherence to use of surgical or P2 mask†	0.26 (0.09–0.77)	0.015‡			
Adherence to use of surgical mask†				0.27 (0.06–1.24)	0.09
Adherence to use of P2 mask†				0.24 (0.05–1.08)	0.06
No. adults	1.07 (0.66–1.71)	0.80		1.06 (0.66–1.71)	0.80
No. siblings	0.86 (0.55–1.35)	0.52		0.86 (0.55–1.35)	0.52
Index patient <5 y of age	0.88 (0.41–1.89)	0.75		0.88 (0.41–1.89)	0.74
Frailty§		0.005‡			0.004‡
2-d incubation period					
Adherence to use of surgical or P2 mask†	0.32 (0.11–0.98)	0.046‡			
Adherence to use of surgical mask†				0.18 (0.02–1.38)	0.099
Adherence to use of P2 mask†				0.45 (0.12–1.62)	0.22
No. adults	1.13 (0.71–1.81)	0.60		1.14 (0.71–1.82)	0.59
No. siblings	0.80 (0.51–1.27)	0.34		0.80 (0.50–1.27)	0.34
Index patient <5 y of age	1.02 (0.46–2.24)	0.96		1.02 (0.47–2.25)	0.95
Frailty§		0.004‡			0.004‡

*ILI, influenza-like illness; CI, confidence interval. †Time-dependent variable. ‡p<0.05 significant (indicates that the outcome for 1 person is correlated with the outcome of other persons in the household). §This term measures if the clustering of subjects in households is relevant to quantify the risk of ILI infection.

## Discussion

We present the results of a prospective clinical trial of face mask use conducted in response to an urgent need to clarify the clinical benefit of using masks. The key findings are that <50% of participants were adherent with mask use and that the intention-to-treat analysis showed no difference between arms. Although our study suggests that community use of face masks is unlikely to be an effective control policy for seasonal respiratory diseases, adherent mask users had a significant reduction in the risk for clinical infection. Another recent study that examined the use of surgical masks and handwashing for the prevention of influenza transmission also found no significant difference between the intervention arms (*12*).

Our study found that only 21% of household contacts in the face mask arms reported wearing the mask often or always during the follow-up period. Adherence with treatments and preventive measures is well known to vary depending on perception of risk (*27*) and would be expected to increase during an influenza pandemic. During the height of the SARS epidemic of April and May 2003 in Hong Kong, adherence to infection control measures was high; 76% of the population wore a face mask, 65% washed their hands after relevant contact, and 78% covered their mouths when sneezing or coughing (*28*). In addition, adherence may vary depending on cultural context; Asian cultures are more accepting of mask use (*29*). Therefore, although we found that distributing masks during seasonal winter influenza outbreaks is an ineffective control measure characterized by low adherence, results indicate the potential efficacy of masks in contexts where a larger adherence may be expected, such as during a severe influenza pandemic or other emerging infection.

We estimated that, irrespective of the assumed value for the incubation period (1 or 2 days), the relative reduction in the daily risk of acquiring a respiratory infection associated with adherent mask use (P2 or surgical) was in the range of 60%–80%. Those results are consistent with those of a simpler analysis in which persons were stratified according to adherence (Technical Appendix ). We emphasize that this level of risk reduction is dependent on the context, namely, adults in the household caring for a sick child after exposure to a single index case. We urge caution in extrapolating our results to school, workplace, or community contexts, or where multiple, repeated exposures may occur, such as in healthcare settings. The exact mechanism of potential clinical effectiveness of face mask use may be the prevention of inhalation of respiratory pathogens but may also be a reduction in hand-to-face contact. Our study could not determine the relative contributions of these mechanisms. In this study, it is only possible to talk about a statistical association between adherent mask use and reduction in the risk of ILI-infection. The causal link cannot be demonstrated because adherence was not randomized in the trial. Although we found no significant difference in handwashing practices between adherent and non-adherent mask users, it is possible that adherent mask use is correlated with other, unobserved variables that reduce the risk of infection. Further work will therefore be needed to definitively demonstrate that adherent mask use reduces the risk of ILI-infection.

In our study, fit testing for P2 masks was not conducted because this is unlikely to be feasible in the general community during a pandemic. As such, we felt it was more appropriate to determine the efficacy of non–fit-tested masks. We found no difference in adherence between P2 and surgical masks, an important finding, as there is a common belief among healthcare workers that P2 masks are less comfortable. The size of the study did not permit conclusive comparison of the relative efficacy of P2 masks and surgical masks. Given the 5- to 10-fold cost difference between the 2 mask types, quantifying any difference in efficacy between surgical masks and particulate respirators remains a priority that needs to be addressed by a larger trial.

A possible limitation of the study is that some adults may have been incubating infection at the time of enrollment. However, this effect would have biased the results toward the null in the intention-to-treat analysis. The survival analysis explicitly accounted for the existence of a fixed incubation period and incubating infections at the time of enrollment. A potential alternative study design would be to enroll participants from asymptomatic households, do follow-up for development of infection, and then immediately intervene with masks. For such a design, given that only 15%–20% of closely exposed adults will develop illness after exposure to an ill child, thousands of households (rather than hundreds) would be required to afford the same study power. In addition, such a design would have been fraught with underascertainment of incident infections and delayed implementation of mask intervention. We believe ours is a more efficient design. A further limitation is that some parents may have acquired infection outside the home. We identified 5 child–parent pairs with discordant viral infections. The randomization process should have ensured that outside exposure was equally distributed between arms, and this effect would have biased the results toward the null.

In retrospect, relying on laboratory-confirmed cases as the primary outcome may have been unrealistic for a study of this size. ILI in enrolled adults was 17.1%, but laboratory confirmation was modest; the virus was identified in only 34.7% of adult ILI cases (the rate of laboratory diagnosis in children was high at 63.8%). However, even intention-to-treat analysis using ILI outcome shows no significant difference between the groups. We used self-reporting to determine adherence; previous research indicates that patient self-reporting is more reliable than judgments by doctors or nurses when compared against urine drug levels (*30*). In addition, the significant association between adherence and clinical protection provides internal validation of self-reporting as a measure.

An important aspect of this study is that we included respiratory viruses other than influenza. Although these viruses may differ in their relative dependence (accurate quantitation of this relativity is uncertain for the various viruses) on different transmission mechanisms (i.e., large droplet, aerosol, or fomite), all are transmitted by the respiratory route. Therefore, face mask use should have some effect on virus transmission (e.g., interference with hand-nose contact), given that participants in all arms of the study received the same infection control advice. In addition, we argue that assessing multiple respiratory viruses allows our results to be generalized more broadly to other infections, including new respiratory viruses that may emerge in the future. Conversely, the low rate of confirmed influenza A or B infection (18.4%) in the study could mean that our findings are not directly applicable to a scenario in which influenza predominates. If influenza is more likely than the other viruses in our study to be transmitted by the respiratory route, the prevalence of mixed infections would tend to bias our results toward the null. However, it is possible that a pandemic strain may have different transmission characteristics than seasonal strains as demonstrated by attack rates in different age groups in pandemics compared with seasonal outbreaks and by the detection of influenza virus in different clinical samples in human influenza virus A (H5N1) cases.

Results of our study have global relevance to respiratory disease control planning, especially with regard to home care. During an influenza pandemic, supplies of antiviral drugs may be limited, and there will be unavoidable delays in the production of a matched pandemic vaccine (*31*). For new or emerging respiratory virus infections, no pharmaceutical interventions may be available. Even with seasonal influenza, widespread oseltamivir resistance in influenza virus A (H1N1) strains have recently been reported (*32*). Masks may therefore play an important role in reducing transmission.

## Supplementary Material

Technical Appendix Face Mask Use and Control of Respiratory Virus Transmission in Households
