# Supplementary material for: Face Mask Use and Control of Respiratory Virus Transmission in Households
Source: Emerg Infect Dis. 2009 Feb;15(2):233–41. doi: 10.3201/eid1502.081167 (PMC2662657; doi:10.3201/eid1502.081167)
Supplement: Technical Appendix — Face Mask Use and Control of Respiratory Virus Transmission in Households [file 08-1167_Techapp-s1.pdf]

Face Mask Use and Control of Respiratory Virus Transmission in Households

Technical Appendix

We present results of a simpler analysis where participants were stratified according to the degree of compliance. Full compliance was defined as when parents reported wearing the mask “all” or “most” of the time (on a 5-point Likert scale) for the first consecutive 5 days. Partial compliance was further defined by the number of consecutive days ($\geq$3 days or $\leq$2 days) from the 1st day of recruitment when participants reported consistently wearing the mask. Table 6 shows the rates of virus isolation and clinical illness by levels of compliance.

We emphasize that data in the Technical Appendix Table should be interpreted with caution as it is possible that adherence is modified once persons have symptoms. For example, adults who plan to comply may stop doing so after they become sick on day 1. If this is the case, the group of “adherent users for 5 days” would select those who wanted to comply and did not get infected, leading to overestimation of the effect of adherent mask use. Note that such a bias cannot occur in the survival analysis presented in Table 5 in the main text, because this table shows the impact of daily adherent use on the daily risk of infection in the days before symptom onset (i.e., this analysis does not rely on adherence status after symptom onset).

Technical Appendix Table. Rates of virus isolation and clinical illness by levels of compliance (RR for all masks, control + non-adherent as reference)*

Variable	Control group, no. (total)	Surgical mask users, no. (total)	P2 mask users, no. (total)	All mask users, no. (total)	RR	95% CI	p value
Fully adherent							
Clinical illness	NA	0/18	1/12	1/30	0.16	0.02–1.14	<0.05
Laboratory confirmed		0/18	1/12	1/30	0.63	0.08–4.79	0.65
Adherent ≥3 d							
Clinical illness	NA	3/36	3/36	6/72	0.42	0.18–0.95	<0.05
Lab confirmed		1/36	2/36	3/72	0.82	0.23–2.69	0.77
Adherent ≤2 d	NA						
Clinical illness		8/19	3/25	11/44	1.25	0.69–2.26	0.46
Lab confirmed		4/19	1/25	5/44	2.25	0.79–6.37	0.12
Non-adherent							
Clinical illness	NA	10/39	8/31	18/70			
Lab confirmed		1/39	5/31	6/70			
Controls							
Clinical illness	16/100	NA	NA	NA			
Lab confirmed	3/100						
Control + non-adherent	34/170						
	9/170						
Total							
Clinical illness		21/94	14/92	35/186			
Lab confirmed		6/94	8/92	14/186			

*RR, relative risk; CI, confidence interval.
